# Was wurde eigentlich aus Prialt®? Beobachtungsstudie über den Langzeiteinsatz von Ziconotid in der Behandlung chronischer Schmerzen

**DOI:** 10.1007/s00482-021-00531-y

**Published:** 2021-01-28

**Authors:** Denise Löschner, Rebecca Dries, Rolf Kalff, Jan Walter, Rupert Reichart

**Affiliations:** Klinik und Poliklinik für Neurochirurgie, Universitätsklinikum Jena, Am Klinikum 1, 07747 Jena, Deutschland

**Keywords:** Intrathekale Schmerztherapie, Ziconotid, Chronischer Schmerz, Schmerzpumpe, Nichtopioidanalgetika, Intrathecal pain management, Ziconotide, Chronic pain, Intrathecal pumps, Non-opioid analgesics

## Abstract

**Hintergrund:**

Prialt® ist seit Februar 2005 von der europäischen Arzneimittelbehörde zugelassen und ist neben Morphin das einzige Analgetikum, welches über die offizielle Marktzulassung in der intrathekalen Schmerztherapie verfügt. Da es nicht über Opioidrezeptoren wirkt, galt es zum Zeitpunkt der Markteinführung als nebenwirkungs- und risikoärmer in der Behandlung chronischer Schmerzen als Morphin. Trotzdem gilt es noch heute als Orphan Drug und Studien über den Langzeiteinsatz und hierunter aufgetretene Nebenwirkungen sind rar.

**Fragestellung:**

Welchen Stellenwert nimmt Prialt® verglichen mit anderen intrathekal verabreichten Analgetika ein? Wie wirken sich die Startdosis und die Geschwindigkeit der Aufdosierung auf die schmerzlindernde Wirkung und das Auftreten von Nebenwirkungen in der Langzeittherapie aus?

**Material und Methoden:**

Zum einen wurden anhand von Arztbriefen retrospektiv alle Patienten erfasst, die zwischen Februar 2005 und dem Ende des Beobachtungszeitraums im Oktober 2018 Ziconotid in Monotherapie in der Neurochirurgie des Universitätsklinikums Jena erhielten. Zum anderen wurden diese Patienten anhand eines erstellten Fragebogens hinsichtlich ihrer Erfahrung mit Ziconotid befragt.

**Ergebnisse:**

Bei allen zwölf in die Studie eingeschlossenen Teilnehmern kam es zu mindestens einer Arzneimittelnebenwirkung. Am häufigsten wurde über Vergesslichkeit und Sensibilitätsstörungen mit jeweils 25 % berichtet. Ein Drittel der Patienten musste die Behandlung aufgrund von Nebenwirkungen beenden. Die mittlere Initialdosis betrug 1,98 µg/Tag.

**Diskussion:**

Trotz leitliniengerechter Behandlung hat sich Ziconotid am Universitätsklinikum Jena nicht gegen Morphin und andere Opioidanalgetika in der intrathekalen Schmerztherapie durchgesetzt. Die Gründe hierfür sind vielfältig, wobei die enge therapeutische Breite, das häufige Auftreten von Nebenwirkungen und die schwierige therapeutische Handhabung, vor allem im ambulanten Setting, von besonderer Bedeutung sind.

## Abstract (en)

**Background:**

Prialt® was approved by the European Medicine Agency in February 2005. Besides morphine, it is the only analgesic approved for long-term intrathecal infusion in the treatment of chronic pain. As it does not bind to opioid receptors, its use in the treatment of chronic pain seemed to be safer and to lead to less adverse events compared with morphine. However, it is an orphan drug and studies of its long-term use are rare.

**Questions:**

What role does Prialt® play in the treatment of chronic pain compared with other analgesics given intrathecally? What impact do the initial dose and the rate of infusion have on the analgesic effect and on the incidence of side effects?

**Material and methods:**

Medical reports were used to identify all patients receiving ziconotide monotherapy from February 2005 to the end of the analysis period in October 2018 in our department. Furthermore, a questionnaire was created and given to the patients to find out more about their experience with ziconotide.

**Results:**

The study included 12 patients, all of whom suffered from at least one adverse event. The most common adverse events were forgetfulness and paraesthesia, each affecting 25% of the patients. One third of the patients discontinued ziconotide therapy due to severe adverse events. The mean initial dose was 1.98 µg/day.

**Discussion:**

Ziconotide was used at the Jena University Hospital according to the latest guidelines. Nevertheless, morphine and other opioid analgesics are still more frequently used in the intrathecal management of chronic pain. There are various reasons for this, but the narrow therapeutic index, the high incidence of adverse events, and the difficulties in finding the right dose are among the most important.

„Ziconotid – der Maßstab in der modernen intrathekalen Schmerztherapie“ lautete der Titel eines Workshops anlässlich des 20. Deutschen interdisziplinären Schmerz- und Palliativkongresses im Jahr 2009. Zehn Jahre später zeigt die Praxis der intrathekalen Schmerztherapie ein anderes Bild. Welchen Stellenwert nimmt Ziconotid, das unter dem Handelsnamen Prialt® verkauft wird, heutzutage in der Therapie chronischer Schmerzen ein und mit welchen Nebenwirkungen ist in der Langzeitbehandlung zu rechnen? In einer retrospektiven Beobachtungsstudie über einen Zeitraum von 13 Jahren wurde nach Antworten gesucht.

## Hintergrund

Neben Morphin sind Ziconotid (Handelsname Prialt®) und Baclofen aktuell die einzigen Medikamente, welche von der amerikanischen Lebensmittelüberwachungs- und Arzneimittelbehörde und der Europäischen Arzneimittelagentur für die intrathekale Therapie zugelassen sind. Letzteres verfügt jedoch nur bei Schmerzen ausgelöst durch Spastiken über die offizielle Marktzulassung [1]. Während Morphin bereits 1979 erstmalig Patienten mit anderweitig nicht kontrollierbaren Schmerzen intrathekal verabreicht wurde [26], verfügt Ziconotid erst seit Februar 2005 über die Zulassung auf dem europäischen Markt [20]. Es handelt sich bei Ziconotid um das synthetische Derivat eines Peptids, welches von der pazifischen Zauberkegelschnecke *Conus magus* zum Töten der Beute produziert wird. Seine Wirkung entfaltet Ziconotid, indem es spannungsabhängige N‑Typ-Kalziumkanäle blockiert, die sich in hoher Konzentration im Hinterhorn des Rückenmarks befinden. Dort sind sie maßgeblich an der Weiterleitung eines Schmerzreizes an das nachgeschaltete Neuron beteiligt [15, 20, 27]. Ziconotid galt als die „*pri*mary *alt*ernative“ zu Morphin, woraus auch dessen Handelsname Prialt® abgeleitet ist [19]. Da Ziconotid gerade nicht über Opioidrezeptoren wirkt, ist mit keiner Toleranzentwicklung zu rechnen [15]. Expertengremien empfehlen daher Morphin und Ziconotid als gleichwertige Erstlinientherapie in der Behandlung chronischer Schmerzen mittels i.t.-Schmerzmittelpumpen. Sofern keine Kontraindikationen vorliegen, sollte Ziconotid sogar dem Morphin in der Behandlung nichttumorassoziierter Schmerzen vorgezogen werden [6]. Trotz der Empfehlung zählt Ziconotid weiterhin zu den Orphan Drugs und im klinischen Umfeld ist der Einsatz von Morphin nach wie vor der Goldstandard [5]. Dies liegt unter anderem an der engen therapeutischen Breite von Ziconotid und den oftmals therapielimitierenden Nebenwirkungen [23]. In drei randomisierten, kontrollierten Studien litten zwischen 92,9 und 97,2 % der mit Ziconotid behandelten Teilnehmer unter mindestens einer unerwünschten Arzneimittelnebenwirkung. Diese waren vornehmlich zentralnervöser Art [17, 22, 24]. Laut Herstellerangaben sind die am häufigsten zu erwartenden Nebenwirkungen Schwindel (42 %), Übelkeit (30 %), Verwirrung (25 %), Nystagmus (23 %), Gangstörung (16 %), Verschwommensehen (14 %), Gedächtnisprobleme (13 %), Asthenie (13 %), Kopfschmerz (12 %), Erbrechen (11 %) und Somnolenz (10 %; [8]). Fallberichte beschreiben darüber hinaus einen Zusammenhang mit erhöhter Suizidalität, auch ohne psychiatrische Vorerkrankung [14]. Für das Auftreten von unerwünschten Arzneimittelnebenwirkungen ist weniger die absolut applizierte Dosis entscheidend, sondern vielmehr die Geschwindigkeit der Aufdosierung [7, 28]. Laut Herstellerangaben sollte mit einer Dosis von 2,4 µg/Tag begonnen werden und diese nicht häufiger als 2‑ bis 3‑mal pro Woche um weitere 2,4 µg/Tag erhöht werden, entsprechend dem Motto „start low, go slow“. Mit dem Eintreten einer analgetischen Wirkung ist ab einer Tagesdosis von 6,0 µg/Tag zu rechnen. Als Maximaldosis gilt in Europa eine Dosis von 21,6 µg/Tag und in den USA eine Dosis von 19,2 µg/Tag [8, 9]. Bis heute gibt es keine Möglichkeit, Ziconotid zu antagonisieren. Treten Arzneimittelnebenwirkungen auf, können diese symptomatisch behandelt werden oder aber die Ziconotidgabe muss unmittelbar gestoppt werden. Das Abklingen der Symptome geschieht in der Regel innerhalb von 24 h, kann jedoch auch mehrere Wochen dauern [7, 20, 28].

## Methoden

### Patientenkollektiv.

In die Beobachtungsstudie wurden alle Personen eingeschlossen, die zwischen Februar 2005 und Oktober 2018 mindestens 1‑mal Ziconotid in Monotherapie über die neurochirurgische Abteilung des Universitätsklinikums Jena erhalten haben. Ziconotid wurde in Jena erstmals im April 2007 angewendet.

### Untersuchungsmaterialien.

Die klinischen Daten stammen aus den Entlassungsbriefen und den Befüllungsprotokollen der Pumpen. Zusätzlich wurde ein Patientenfragebogen ausgehändigt, in dem die Patienten vor allem hinsichtlich der Zufriedenheit mit dem Medikament und aufgetretener Nebenwirkungen befragt wurden. Acht Patienten nahmen an der Befragung teil. Gründe für die fehlende Teilnahme waren u. a. Versterben, Umzug mit fehlender Möglichkeit zur späteren Kontaktaufnahme, aber auch Ablehnung seitens der Patienten.

## Ergebnisse

**Figure 1:**
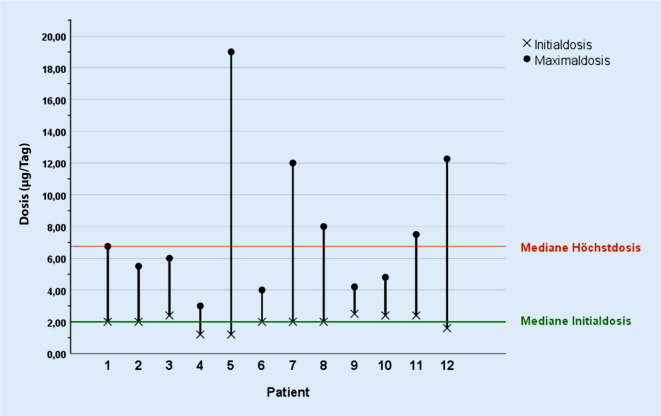

Sechs Männer und sechs Frauen wurden in der Neurochirurgie Jena innerhalb des Beobachtungszeitraums mit Ziconotid behandelt. Bei Therapiebeginn waren sie zwischen 44 und 71 Jahre (Mittelwert: 58,58 Jahre) alt. Bei zehn der zwölf Patienten lag die Diagnose chronischer Schmerz (ICD R52.1 und ICD R52.2) vor. Zwei Patienten litten unter einer Radikulopathie im Lumbalbereich (ICD M54.16). Ursächlich für die chronischen Schmerzen waren hierbei in 69,2 % ein oder mehrere Eingriffe an der Wirbelsäule, in deren Folge die Schmerzen nicht abnahmen oder sich sogar verschlimmerten. Dieses Krankheitsbild wird auch als „failed back surgery syndrome“ (FBSS) bezeichnet [2]. Die Initialdosis betrug zwischen 1,2 und 2,4 µg/Tag mit einem Mittelwert von 1,98 µg/Tag. Der Median lag bei 2 µg/Tag. Die individuelle Höchstdosis betrug zwischen 3 und 19 µg/Tag mit einem Mittelwert von 7,75 µg/Tag und einem Median bei 6,36 µg/Tag (Abb. 1). Retrospektiv wurde ebenfalls die Durchschnittsdosis pro Tag über einen Zeitraum von 5 Jahren ermittelt. Diese lag am Tag 14 bei 3,1 µg/Tag, nach der vierten Woche bei 3,9 µg/Tag, nach der achten Woche bei 3,6 µg/Tag und nach der zwölften Woche bei 3,8 µg/Tag. Nach sechs Monaten erhielten die Patienten im Durchschnitt eine Dosis von 4,0 µg/Tag. Fünf Patienten erhielten Ziconotid auch noch nach einem (4,5 µg/Tag) und nach zwei Jahren (5,5 µg/Tag). Vier Patienten gingen in die Betrachtung der Durchschnittsdosis nach drei (8,0 µg/Tag), vier (10,6 µg/Tag) und fünf Jahren (9,0 µg/Tag) ein. Nach dem sechsten und siebten Jahr lag die mittlere Dosis bei 10,2 µg/Tag bzw. 10,3 µg/Tag (Tab. 1). Der Behandlungszeitraum mit Ziconotid bis einschließlich 31.10.2018 variierte zwischen 23 und 3709 Tagen. Die mittlere Behandlungsdauer lag bei 1219 Tagen. Prialt® wurde klassischerweise als Drittlinientherapie eingesetzt (50,0 %). Drei Patienten (25 %) erhielten Ziconotid als Zweitlinienanalgetikum in der intrathekalen Schmerztherapie. Nur ein Patient erhielt Ziconotid als First-in-pump-Therapie.

**Table 1:** 

	Woche 2	Woche 4	Woche 8	Woche 12	Monat 6	Monat 12	Jahr 2	Jahr 3	Jahr 4	Jahr 5	Jahr 6	Jahr 7
	*n* = 11	*n* = 11	*n* = 9	*n* = 9	*n* = 7	*n* = 5	*n* = 5	*n* = 4	*n* = 4	*n* = 4	*n* = 3	*n* = 3
Dosis (µg/Tag)												
Mittlere Dosis	3,1	3,9	3,6	3,8	4,0	4,5	5,5	8,0	10,6	9,0	10,2	10,3
Minimaldosis	1,5	1,5	1,7	2,0	2,7	3,0	3,0	3,5	5,7	5,7	5,3	5,5
Maximaldosis	6,0	10,0	6,0	8,0	6,0	9,1	9,5	12,6	17,8	14,7	14,7	15,4

**Figure 2:**
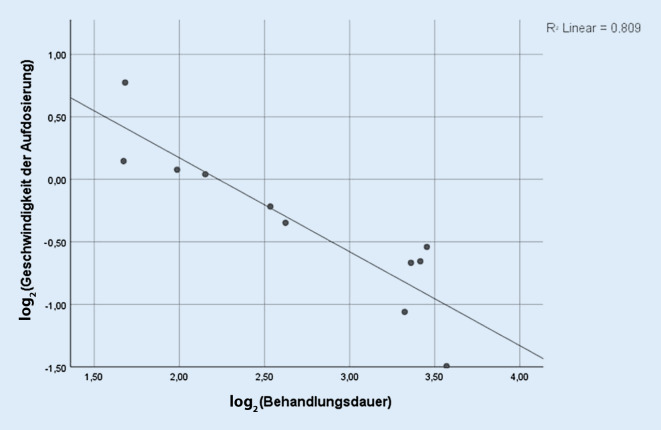

Für das Auftreten unerwünschter Arzneimittelnebenwirkungen spielt nachweislich die Geschwindigkeit der Aufdosierung eine entscheidende Rolle [7, 28]. Geht man nun vereinfacht davon aus, dass weniger Nebenwirkungen mit einer längeren Gesamtbehandlungsdauer einhergehen, wurde versucht, einen Zusammenhang zwischen der Geschwindigkeit der Aufdosierung und der Dauer der Ziconotidbehandlung aufzuzeigen (Abb. 2). Diese berechnet sich bei jedem der untersuchten Patienten aus der Geschwindigkeit der Aufdosierung bis zur persönlichen Höchstdosis in µg/28 Tage im Verhältnis zur gesamten Dauer der Prialt®-Therapie. Mit einem R^2^ (Determinationskoeffizient) von 0,809 ist ein linearer Zusammenhang zwischen Geschwindigkeit der Aufdosierung und Behandlungsdauer naheliegend. Eine langsamere Höherdosierung von Ziconotid scheint mit einer längeren Therapiedauer einherzugehen.

### Nebenwirkungen unter der Ziconotidbehandlung.

Es wurde zunächst einmal zwischen den in den Arztbriefen dokumentierten und gesicherten Nebenwirkungen und den von den Patienten anhand des Fragebogens angegebenen Ereignissen unterschieden. Eine schwere Arzneimittelnebenwirkung lag vor, wenn diese das Patientenleben unmittelbar bedrohte und/oder mit einem erneuten oder verlängerten Krankenhausaufenthalt verbunden war. Laut Arztbriefen litten sechs der zwölf Patienten unter mindestens einer schweren Arzneimittelnebenwirkung. Bei zwei Patienten trat ein akuter Harnverhalt auf und jeweils einmal wurden Halluzinationen, eine motorische Aphasie, schwerwiegende psychische Veränderungen im Sinne einer zunehmenden Vergesslichkeit und Dyspnoe beobachtet. Zehn von zwölf Patienten (83,3 %) berichteten über mindestens eine milde bis moderate Medikamentennebenwirkung. Insgesamt wurden 36 solcher Ereignisse in den Arztbriefen dokumentiert. 23 der 36 Nebenwirkungen (63,9 %) waren psychischen oder zentralnervösen Ursprungs. Am häufigsten wurde über Vergesslichkeit und das Auftreten von Sensibilitätsstörungen berichtet. Jeweils drei von zwölf Patienten (25 %) litten darunter. Schwindel, Sehstörungen, Sprechstörungen, Verwirrtheit, Konzentrationsstörungen, Miktionsprobleme, Gewichtszunahme und Übelkeit betrafen jeweils zwei Patienten (16,7 %). Im untersuchten Patientenkollektiv schien Suizidalität als Nebenwirkung keine Relevanz zu haben.

## Diskussion

Das untersuchte Patientenkollektiv unterschied sich hinsichtlich Geschlechterverteilung und Patientenalter nicht wesentlich von den in Vergleichsstudien beschriebenen Werten über den Langzeiteinsatz von Ziconotid [3, 10, 16, 25]. Das „failed back surgery syndrome“ ist in vergleichbaren Studien, soweit es ihnen zu entnehmen ist, ebenso die am häufigsten zugrunde liegende Erkrankung [3, 16]. Die in Jena gewählte mittlere Startdosis von 1,98 µg/Tag entspricht den Herstellerangaben. Neuere Empfehlungen legen jedoch eine weitaus geringere Startdosis von maximal 0,5 µg/Tag und eine Erhöhung um weitere 0,5 µg/Tag maximal 1‑mal pro Woche nahe [11]. Vor dem Hintergrund, dass unter Ziconotid eine Analgesie erst ab einer Dosis von 6 µg/Tag zu erwarten ist, würde sich die Aufdosierungsphase über knapp drei Monate erstrecken. Es bleibt sicher kritisch zu hinterfragen, wie sich dieses Therapiekonzept im klinischen Umfeld umsetzen lässt und wie es vor allem auch von Seiten des Patienten toleriert wird. Ebenso stellt sich die Frage, ob dieses Therapieschema unter medizinischen Gesichtspunkten sinnvoll und kostendeckend zu gleich ist. Nichtsdestotrotz geht ein langsameres Aufdosierungsschema mit weniger Nebenwirkungen einher, weshalb in Zukunft geringere Startdosen wünschenswert sind.

Die mittlere, in dieser Beobachtungsstudie eingesetzte Ziconotiddosis zeigte bis zum Ende des vierten Behandlungsjahrs eine steigende Tendenz, um sich dann auf einem Niveau von etwa 10 µg/Tag zu halten (Tab. 1). Es wurden allerdings auch nur vier Patienten mehr als vier Jahre mit Prialt® behandelt. Eine Toleranzentwicklung konnte auch in dieser Datenanalyse nicht beobachtet werden. Ein Patient wird bis dato seit mehr als elf Jahren erfolgreich mit Ziconotid behandelt und das bei einer seit Jahren konstanten Dosis von ca. 6 µg/Tag. Dieser Patient ist besonders hervorzuheben, da es in der Literatur bislang keine Informationen über einen derart langen Behandlungszeitraum gibt.

Unsere Beobachtungen legen nahe, dass es einen linearen Zusammenhang zwischen der Geschwindigkeit der Dosiszunahme und der Gesamtbehandlungsdauer gibt: Je langsamer die Dosis gesteigert wurde, desto länger wurden die Patienten insgesamt mit Ziconotid therapiert. Es bleibt jedoch die Frage offen, ob die längere Behandlungsdauer unter geringerer Aufdosierungsgeschwindigkeit wirklich auf eine bessere Wirksamkeit und Verträglichkeit von Ziconotid zurückzuführen ist oder ob sich die Patienten aufgrund der längeren Austestungsphase schlichtweg in längerer ärztlicher Behandlung befanden. Eine sich über Monate erstreckende Aufdosierung bis zu einer wirksamen Dosis kann auch eine erfolgreiche und vor allem lange Schmerztherapie vortäuschen. Die Patienten erfahren in dieser Zeit unter Umständen keine zufriedenstellende Schmerzlinderung und sind daher zusätzlich auf eine analgetische Komedikation und konservative schmerztherapeutische Maßnahmen angewiesen.

Obwohl Morphin und Ziconotid beide als gleichwertige Erstlinientherapie empfohlen werden [6], wurde Ziconotid in der neurochirurgischen Klinik Jena typischerweise erst als Drittlinientherapeutikum eingesetzt. Der Stellenwert von Ziconotid in der intrathekalen Therapie in Jena sollte auch vor dem Hintergrund kritisch betrachtet werden, dass laut einer Studie mit über 90 Teilnehmern Prialt® als First-in-pump-Therapie zu einer signifikant besseren Schmerzreduktion führte, verglichen mit Patienten, die vorher bereits ein anderes intrathekales Analgetikum erhalten hatten [3]. Im Gegensatz zu Ziconotid wird Morphin bereits seit Beginn der intrathekalen Schmerztherapie in den 1980er-Jahren angewendet und es ist damit das am häufigsten in Studien untersuchte Pharmakon für diesen Applikationsweg [13, 18, 23]. Das Nebenwirkungsspektrum ist hierbei gut untersucht, entspricht weitestgehend dem der oralen Opiateinnahme, wobei viele der bekannten Nebenwirkungen selbstlimitierend sind oder sich durch die Einnahme zusätzlicher Medikamente gut behandeln lassen [12]. Ein großer Vorteil ist zudem, dass Morphin mit Naloxon antagonisiert werden kann, während es bis dato keinen Antagonisten zu Ziconotid gibt. Es darf auch nicht unerwähnt bleiben, dass sich die Kosten für eine definierte Tagesdosis Ziconotid auf 59,99 € belaufen (Rechenbeispiel für 12 µg Tagesdosis Prialt® bei Medikamentenkosten von 503 € für 100 mg/ml). Es ist damit über 400-mal teurer als Morphin [21]. Ein Medikamentenwechsel, der hinsichtlich der häufig auftretenden Nebenwirkungen unter stationären Bedingungen erfolgen sollte, und die regelmäßigen Vorstellungen zur Aufdosierung verursachen weitere Kosten [4]. Der Hauptgrund, weshalb Ziconotid heutzutage nur zögerlich eingesetzt wird, ist aber wohl weiterhin dessen enge therapeutische Breite. Hinsichtlich des Auftretens von Nebenwirkungen zeigen unsere Ergebnisse übereinstimmend mit der Literatur, dass sowohl in der Kurzzeittherapie als auch in der Langzeitbehandlung mit Prialt® bei fast allen Patienten mit Nebenwirkungen, vornehmlich zentralnervöser Art, zu rechnen ist. Minimale Unterschiede in der prozentualen Verteilung sind mit Sicherheit auf die Größe des untersuchten Patientenkollektivs zurückzuführen. Während laut Herstellerangaben schwere Nebenwirkungen nur selten sind, war in der aktuellen Analyse jeder zweite Patient davon betroffen [8]. In vier von sechs Fällen hatten die Nebenwirkungen den Therapieabbruch zur Folge. Ob die Nebenwirkungen schlussendlich wirklich auf Ziconotid zurückzuführen waren oder aber die Ursache in der Grunderkrankung, den Nebendiagnosen oder der Komedikation des Patienten lag, blieb häufig offen. So war in einem der beiden von uns dokumentierten Fälle eines akuten Harnverhalts das benigne Prostatasyndrom des Patienten wohl wahrscheinlicher die Ursache der Miktionsprobleme.

## Fazit für die Praxis

- Prialt® ist ein Analgetikum, bei dessen Einsatz mit einer Vielzahl von Nebenwirkungen vor allem zentralnervöser und psychischer Art zu rechnen ist.
- Die richtige Dosisfindung gestaltet sich in der Praxis oft schwierig und erfordert sowohl vom behandelnden Arzt als auch vom Patienten viel Geduld und Mitarbeit. Eine gute Arzt-Patienten-Bindung ist hierbei eine umso wichtigere Voraussetzung für den Therapieerfolg.
- Auch wenn Ziconotid in Studien als First-in-pump-Analgetikum eine bessere Wirksamkeit verglichen mit einer Zweit- oder Drittlinientherapie zeigte, hat es bis jetzt nicht Morphin als das Mittel der ersten Wahl in der intrathekalen Therapie abgelöst.
- Unsere Beobachtungen legen den Schluss nahe, dass es das in naher Zukunft auch nicht tun wird. Dafür ist schon die Handhabung des Medikaments zu schwierig und das Nebenwirkungsprofil übersteigt den therapeutischen Nutzen oftmals deutlich.
